# Evaluation of a metal artifact reduction algorithm in CT studies used for proton radiotherapy treatment planning

**DOI:** 10.1120/jacmp.v15i5.4857

**Published:** 2014-09-08

**Authors:** Karin M. Andersson, Anders Ahnesjö, Christina Vallhagen Dahlgren

**Affiliations:** ^1^ Department of Medical Physics Örebro University Hospital Örebro Sweden; ^2^ Section for Medical Radiation Physics Department of Radiology, Oncology and Radiation Sciences, Uppsala University Uppsala Sweden; ^3^ Skandionkliniken Uppsala Sweden

**Keywords:** metal artifact reduction, radiation therapy, water equivalent thickness, proton range

## Abstract

Metal objects in the body such as hip prostheses cause artifacts in CT images. When CT images degraded by artifacts are used for treatment planning of radiotherapy, the artifacts can yield inaccurate dose calculations and, for particle beams, erroneous penetration depths. A metal artifact reduction software (O‐MAR) installed on a Philips Brilliance Big Bore CT has been tested for applications in treatment planning of proton radiotherapy. Hip prostheses mounted in a water phantom were used as test objects. Images without metal objects were acquired and used as reference data for the analysis of artifact‐affected regions outside of the metal objects in both the O‐MAR corrected and the uncorrected images. Water equivalent thicknesses (WET) based on proton stopping power data were calculated to quantify differences in the calculated proton beam penetration for the different image sets. The WET to a selected point of interest between the hip prostheses was calculated for several beam directions of clinical relevance. The results show that the calculated differences in WET relative to the reference case were decreased when the O‐MAR algorithm was applied. WET differences up to 2.0 cm were seen in the uncorrected case while, for the O‐MAR corrected case, the maximum difference was decreased to 0.4 cm. The O‐MAR algorithm can significantly improve the accuracy in proton range calculations. However, there are some residual effects, and the use of proton beam directions along artifact streaks should only be used with caution and appropriate margins.

PACS numbers: 87.55.D‐, 87.57.cp

## I. INTRODUCTION

In radiotherapy it is standard routine to use X‐ray computed tomography (CT) to provide a basis for planning of the treatment. CT images are used for delineation of targets and organs at risk (OARs), and the Hounsfield units (HU) provide quantitative data for mapping to radiation transport data in dose calculations. A common problem in CT is the occurrence of image artifacts when metallic objects, such as dental fillings or orthopedic prostheses, are present in the patient. The artifacts usually appear as dark and bright streaking zones across the reconstructed image. With artifacts present in the scanned volume, the acquired HU values will map to erroneous interaction properties, which affects the results of the dose calculations and can seriously jeopardize the entire radiotherapy planning process.^1^, ^2^ In the case of radiation therapy with protons, the proton range is highly dependent on the tissue composition, and artifacts in the CT images may introduce unacceptable errors in the calculations of proton ranges and dose distributions.

Li et al.^(3)^ have shown that both the HU accuracy and the visual appearance of target and OARs were improved when using the metal artifact reduction software O‐MAR (Metal Artifact Reduction for Orthopedic Implants) (Philips Healthcare, Andover, MA) for cases where metallic hip prostheses were present. In this paper we describe an evaluation of the O‐MAR algorithm in the context of treatment planning for proton radiation therapy. A hip prostheses phantom was scanned and both O‐MAR corrected images and uncorrected images were evaluated based on the water equivalent thickness (WET) concept.

## II. MATERIALS AND METHODS

The software O‐MAR was installed on a Philips Brilliance Big Bore CT (Philips Healthcare) used at the radiotherapy clinic at the Uppsala University Hospital. CT images of a water phantom, in which metallic hip prostheses could be mounted, were acquired and analyzed. Copies of the images were O‐MAR corrected and compared with the original uncorrected images for the regions outside the metal objects. Images of the water‐filled phantom without the prostheses were used as reference data.

### A. The O‐MAR algorithm

**Figure 1:**
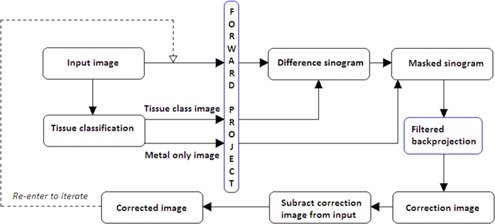
Flow chart of the iterative procedure used in the O‐MAR algorithm for metal artifact reduction. (Adapted from Philips Healthcare white paper.^(4)^)

A flow chart of the O‐MAR algorithm^(4)^ is shown in Fig. 1. HU value thresholds are set to classify an input image into a metal only image and a tissue classified image. The metal only image is defined by assigning all pixels to zero, except the ones categorized as metal. The tissue classified image is defined by setting the pixels with a HU value close to zero to the average HU value of the tissue pixels. The remaining pixels are then defined as nontissue pixels with their HU values unmodified. The original image and the two segmented images are forward projected and a difference sinogram is created by subtracting the tissue classified sinogram from the original image sinogram. All the nonmetal data are removed from the difference sinogram by using the metal only sinogram as a mask. Hence, the resulting sinogram can be interpreted as a “metal only” recording. A correction image, created by back‐projection of the simulated metal recording is then subtracted from the original input image to obtain a corrected image. This corrected image is then used as the input image and the process is iterated until convergence. During the first iteration, the metal data points in the original image sinogram are identified and replaced by interpolated values to emulate tissue.

### B. Phantom imaging

**Figure 2:**
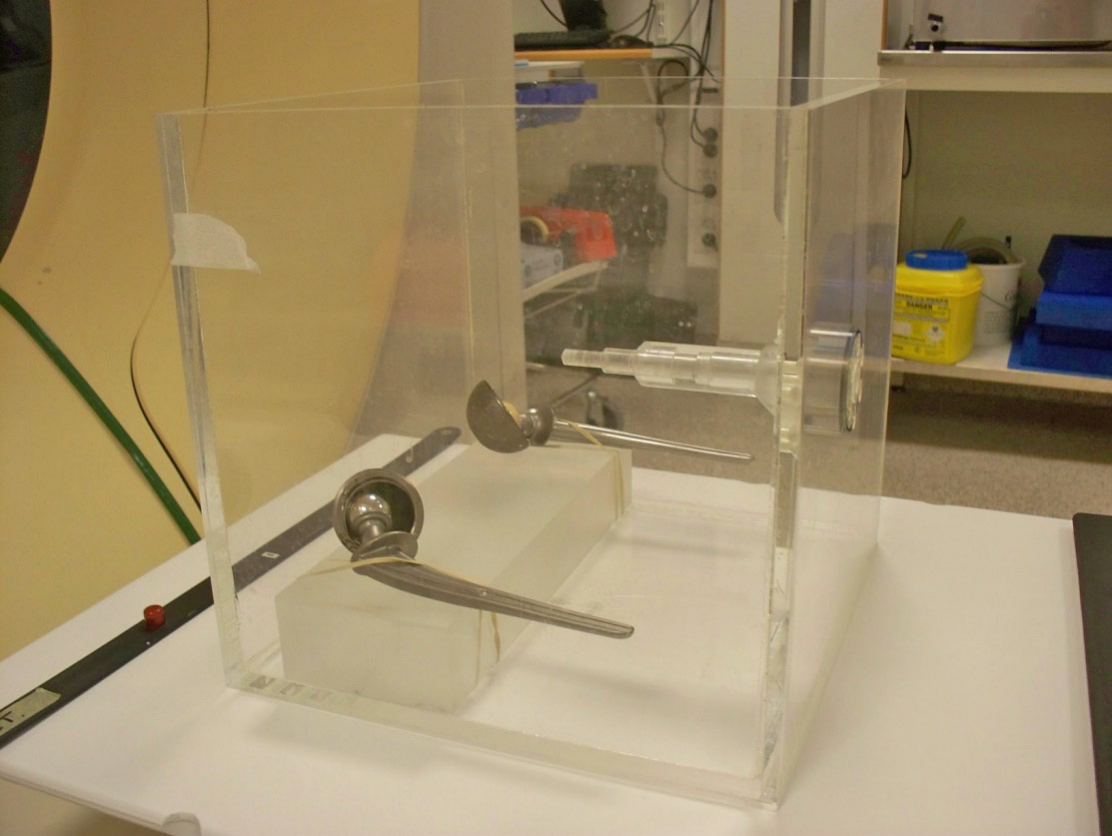
The phantom with hip prostheses placed on a slab of PMMA. During imaging the phantom was filled with water to approximately 23 cm.

An image of the phantom used in the evaluation is shown in Fig. 2. The phantom had a cross section of $30\;\times \;30\;{\text{cm}}^{2}$ and was filled with water to a depth of approximately 23 cm. Two chromium‐cobalt hip prostheses with cups were placed 20 cm apart on a slab of PMMA resting on the bottom of the water phantom. Imaging was done with a helical CT protocol typically used for the pelvic area (120 kVp, 420 mAs and 3 mm slice thickness).

Images were first captured with the prostheses present in the phantom, followed by acquiring reference images with the metal objects removed (i.e., replaced by water). Images with both one and two prostheses present were acquired. The supporting slab of PMMA was present in the phantom during all scans. Images corresponding to the central area of the femoral heads were chosen for the investigation because of its relevance for radiotherapy in the pelvic area.

### C. Calculation of water equivalent thickness

In the imaged phantom, a central position in between the prostheses was chosen. The WET values for proton beams directed to this point were then calculated for the original uncorrected image with prostheses, the O‐MAR corrected images, and the reference images scanned with water replacing the prostheses.

WET is defined as the thickness of water that causes a hypothetical straight line ray to lose the same amount of energy as the beam ray would lose in a medium, *m*, of thickness, $t_{m}$. The WET is in the thin target approximation given by:^(5)^

(1) $$\text{WET}=t_{w}=t_{m}\frac{\rho_{m}{\left(S/\rho \right)}_{m}}{\rho_{w}{\left(S/\rho \right)}_{w}}$$

where $t_{w}$ and $t_{m}$ are the thicknesses of water and the medium corresponding to an equivalent energy loss of the incident protons, $\rho_{w}$ and $\rho_{m}$ are the mass densities of water and the medium, and ${\left(S/\rho \right)}_{w}$ and ${\left(S/\rho \right)}_{m}$ are the mass stopping power values for water and the medium, respectively. Values for the relative stopping power ratio,

(2) $$\rho_{s}=\frac{\rho_{m}{\left(S/\rho \right)}_{m}}{\rho_{w}{\left(S/\rho \right)}_{w}}$$

were taken for lung, adipose, muscle, cartilage, and bone from the tabulation given by Schneider et al.^(6)^ The mapping from HU to relative stopping powers was done in a two‐step procedure. First, following the approach of Schneider et al.^(7)^ and Vanderstraeten et al.^(8)^ for implementing the CT calibration proposed by Schneider et al.,^(6)^ our CT scanner was used to image a phantom of water and PMMA with inserts of various concentrations of ${\text{CaCl}}_{2}$ dissolved in water. These measurements were used to determine the parameters $K^{\text{ph}}/K^{\text{KN}}$ and $K^{\text{coh}}/K^{\text{KN}}$ (following the nomenclature of the above references), yielding the values $2.86\;\times \;10^{‐5}$ and $7.20\;\times \;10^{‐4}$, respectively, through a fitting procedure. In a second step, the corresponding HU for the tissues mentioned above were calculated based on the chemical composition and density given by ICRP Report 23^(9)^ using the fitted parameter values. Linear interpolation and, if needed, extrapolation were used to obtain the relative stopping power values. The HU values in the area of the images, which the data in this study corresponds to, were in the range from ‐740 HU to 1370 HU. The relative stopping power values given by Schneider^(6)^ were calculated for 219 MeV protons which, within an energy range from 50 MeV to 250 MeV, are accurate within 1% for cortical bone and 0.5 % for adipose and muscle. Although the WET for a water phantom should equal the geometrical ray length in water, we choose to follow the procedure outlined above as to closer mimic the common clinical situation with a treatment planning system interpreting a scanned image as to represents human tissues instead of phantom materials. Thus, a systematic error is introduced in that WET calculated for HU = 0 not exactly equals the geometrical ray length in water, but instead gives the result for the tissue mapped at HU = 0 (i.e., a mixture of adipose and muscle). This systematic error cancels while scanning and deriving the reference WET for the water phantom through exactly the same calculations procedure instead of using the geometrical distances directly. Furthermore, it allows for using PMMA as part of the phantom, yielding a similar systematic deviation also canceling between the test and the reference data.

The WET value from $r_{0}$ to r is thus defined as:

(3) $$\text{WET}(r_{0}r)=\int_{r_{0}}^{r}\rho_{s}\text{ds}$$

which we calculated using its discrete counterpart

(4) $$\text{WET}(r,r_{0})=\sum_{i=1}^{N}\rho_{s}(r)\cdot \Delta s_{i}=\left|r-r_{0}\right|\frac{1}{N}\sum_{i=1}^{N}\rho_{s}(s_{i})$$

where the index *i* run over all the *N* voxels intersected by the line from $r_{0}$ to r, and $\Delta s_{i}$ is the ray length in the voxel at step *i*.

**Figure 3:**
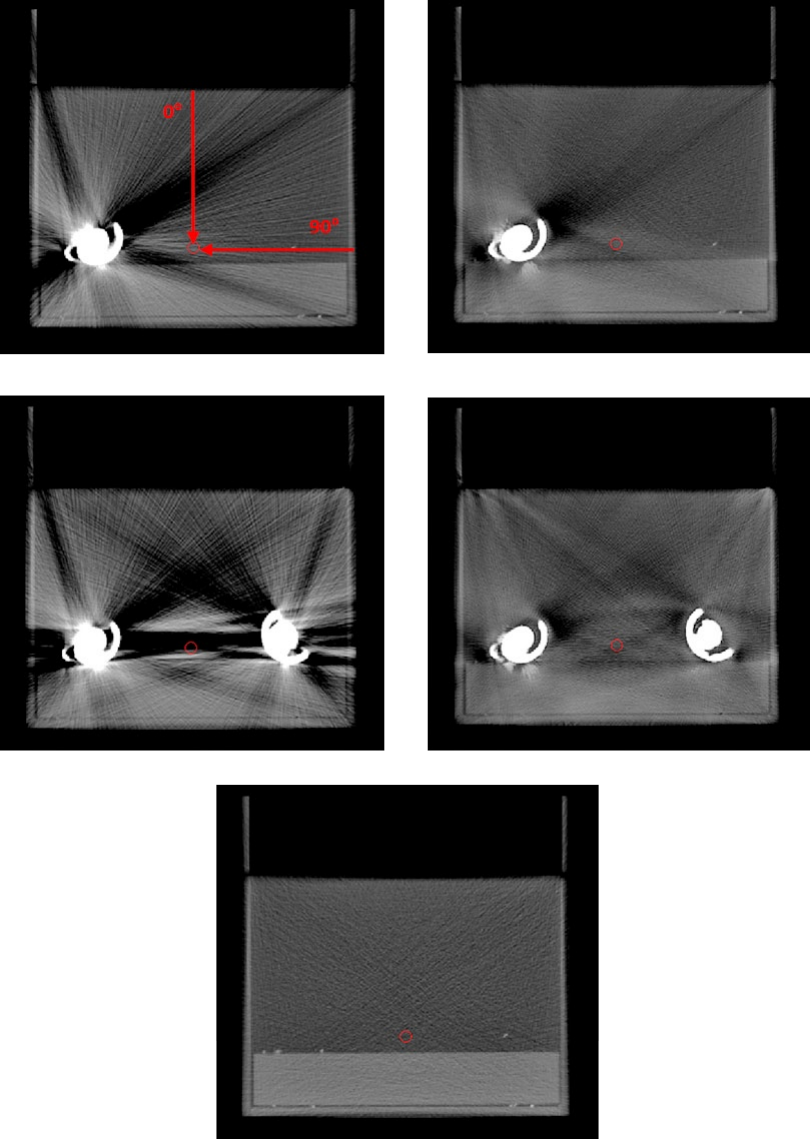
Uncorrected (left) and O‐MAR corrected (right) CT images of the phantom with one hip prosthesis (top) and two hip prostheses (middle). The reference image without any metal present is shown at the bottom. The position of the point of interest, to which the WETs are calculated, is marked by a circle. WET values were calculated from the edge of the phantom to the point of interest at every forth degree from 0° to 360° (0° and 90° are marked in the top left image).

The WET was calculated in MATLAB (MathWorks, Natick, MA) along lines to a point of interest, placed between the prostheses, from the edge of the water phantom. The locations of the points of interests are shown in Fig. 3 in the uncorrected and the O‐MAR corrected CT images of the phantom. The WET was calculated for line directions at every forth degree. The differences in WET, compared to the reference case, were calculated for the uncorrected and the OMAR corrected images. The WET was calculated for the entire path from the edge of the phantom to the point of interest, but also from the edge of the phantom to every single point along the specific ray line. These calculations were made to determine whether the maximum WET difference was found at the end of the path to the point of interest, or at some prior point along the ray lines.

## III. RESULTS & DISCUSSION

**Figure 4:**
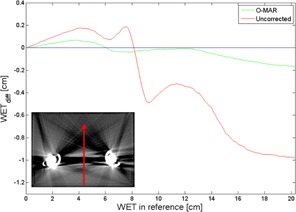
WET differences, along the line marked in the inserted image, for the O‐MAR corrected image and the uncorrected image as functions of the WET at the corresponding position in the reference image. The use of the O‐MAR algorithm led to a substantial decrease of the metal artifact‐induced WET difference. The line of zero WET difference is provided to guide the eye.

**Figure 5:**
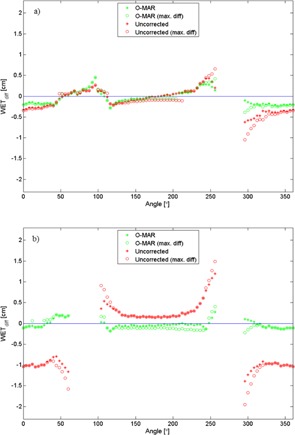
Differences in WET between the reference images and the uncorrected and O‐MAR corrected images, respectively, for the phantom with one prosthesis (a) and two prostheses (b). The WET values are calculated from the edge of the phantom to a point of interest between the prostheses. The WET data corresponding to beam angles intersecting the prostheses are excluded. The lines of zero WET difference are provided to guide the eye.

In Fig. 4, WET differences $\left({\text{WET}}_{\text{diff}}\;=\;{\text{WET}}_{O‐\text{MAR}/\text{Uncorr}}\;‐\;{\text{WET}}_{\text{ref}}\right)$ versus the reference image are shown for a posterior–anterior (PA) beam direction for the O‐MAR corrected image and the uncorrected image. In Fig. 5, the WET difference is given as a function of beam direction. In all cases, the WET difference is calculated from the edge of the phantom to the point of interest. The direction of the path is represented by the angle, where 0° corresponds to the direction from the top of the phantom straight down to the point of interest and 90° corresponds to the direction from the right side of the phantom to the point of interest (see Fig. 3). The maximum WET differences along the paths are also plotted in Fig. 5.

The results show that the O‐MAR algorithm significantly improves the WET values in artifact‐affected CT images, and can thus improve the range accuracy in dose calculations by several millimeters. In Fig. 4, the WET was calculated along a line at 180° across the low‐density artifact between the two hip prostheses. For this case, the maximal WET difference was reached at the most distal depth, about 20 cm into the phantom, where the WET difference was almost 1.0 cm for the uncorrected case. The corresponding WET difference in the O‐MAR case was 0.2 cm.

In the case with two prostheses, the maximum WET difference as a function of angle was 2.0 cm for the uncorrected case and 0.4 cm for the O‐MAR corrected case. Imaging of the unilateral prosthesis phantom showed smaller deviations in WET relative to the reference, but the O‐MAR algorithm generally improved the accuracy.

It is important to apply margins in proton treatment planning to account for range uncertainties. A common distal margin is about 4% of the range.^(10)^ Our results indicate that additional distal and proximal margins may need to be considered, even with the correction methods demonstrated here.

As seen in Fig. 5, the WET differences vary significantly with direction in the image, which means that the accuracy in the calculated proton range will highly depend on the direction of the beam relative to the artifacts. Proton beams applied along streaks results in larger deviations, and should be used with caution and appropriate margins.

Further evaluation of the O‐MAR application could be performed by using a more complex heterogeneous phantom. According to a white paper from Philips, the O‐MAR algorithm may incorrectly modify areas with metal in close proximity to air.^(4)^ Additional information may, therefore, be gained by adding inhomogeneities to the phantom, such as an air cavity simulating rectum, and evaluate the function of the algorithm on such images.

In addition to implants made of chromium‐cobalt, which is the type of prostheses used in this phantom study, titanium and stainless steel are also used as material for hip prostheses. An additional parameter in the evaluation of a metal artifact reduction algorithm could therefore be different hip prostheses material. However, the chromium‐cobalt alloy has a higher attenuation coefficient than titanium and steel^(11)^ and would, as a result, cause larger degradation of the CT images. Hence, artifacts originating from implants of titanium and stainless steel are expected to cause less error in proton treatment planning than chromium‐cobalt implants, but it still is an aspect that would be of interest to investigate further.

Another technique used for reducing the impact of metal artifacts on the CT image‐based dose calculations, apart from a MAR algorithm, is to override the artifact‐affected areas by assigning it to be composed of water or a homogeneous tissue. The override technique is however both time‐consuming and subjective, factors which can be eliminated with automated approaches like O‐MAR corrections.

In this evaluation, a single setting of the CT protocol was used. According to the white paper from Philips,^(4)^ reduction of metal artifacts improves with higher kV and higher mAs values. Consequently, a further investigation of the function O‐MAR with varying CT settings would be of interest.

## IV. CONCLUSIONS

This study confirms, based on WET calculations, that the O‐MAR algorithm has capacity to reduce uncertainties in proton dose calculations when metal artifacts are present in the CT images. The method of comparing WET values, as a way to evaluate the impact of metal artifacts in CT studies used for proton therapy treatment planning, is generic and could easily be generalized to other geometries.

## Supporting information

Supplementary Material
