# Supplementary figures and images for: Evaluation of a metal artifact reduction algorithm in CT studies used for proton radiotherapy treatment planning
Source: J Appl Clin Med Phys. 2014 Sep 8;15(5):112–9. doi: 10.1120/jacmp.v15i5.4857 (PMC5711074; doi:10.1120/jacmp.v15i5.4857)

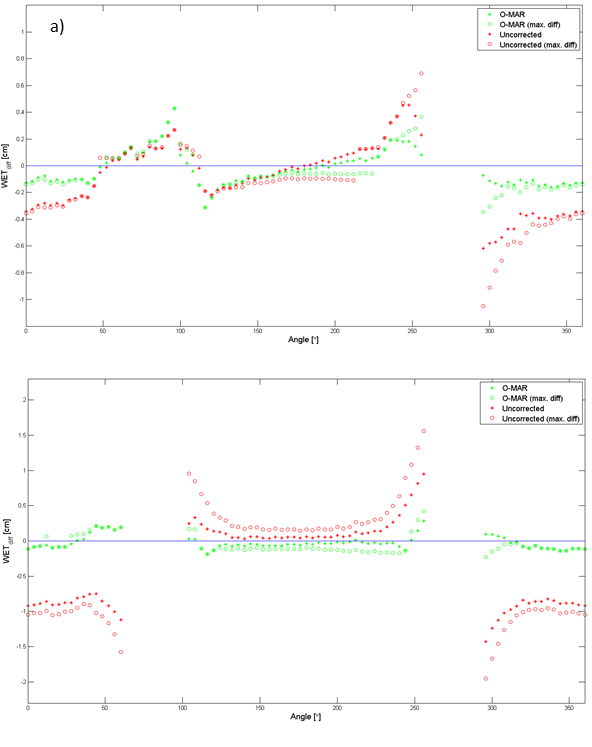

Supplement: Supplementary file 1 — Supplementary Material [file ACM2-15-112-s001.png]
